# How Best to Play the Role of Tumor Deposits in Stage III Colon Cancer?

**DOI:** 10.3389/fonc.2022.860491

**Published:** 2022-02-28

**Authors:** Yunxiao Liu, Hao Zhang, Yuliuming Wang, Chunlin Wang, Huan Xiong, Yang Wang, Haoyu Jing, Xia Jiang, Hanqing Hu, Qingchao Tang, Guiyu Wang

**Affiliations:** Department of Colorectal Surgery, the Second Affiliated Hospital of Harbin Medical University, Harbin, China

**Keywords:** colon cancer, pathological N stage, tumor deposits, prognosis, cancer-specific survival

## Abstract

**Background:**

The purpose of this study is to comprehensively evaluate the prognostic role of tumor deposits (TD) in stage III colon cancer.

**Methods:**

24,600 CC patients with III stage colon cancer were collected from the Surveillance, Epidemiology, and End Result (SEER) database and 618 CC patients from the Second Affiliated Hospital of Harbin Medical University. All patients were divided into development, internal, and external validation cohorts. The combination of positive lymph nodes (PLN) and the status or number of TD was defined as modified pN (mpN) and novel pN (npN). The Cox proportional hazard regression model was used to analyze the relationship between cancer-specific survival (CSS) and mpN or npN. CSS stratified by pN, mpN, and npN was analyzed by the Kaplan–Meier curves. The area under the receiver operating characteristic curve (AUC) was used to demonstrate the predictive abilities of the pN, mpN, and npN stages. The validation cohorts were used to validate the results.

**Results:**

The Cox proportional hazard regression model showed that mpN and npN were an independent prognostic factor for CSS. AUC showed that the predictive accuracy of mpN was better than that of the pN stage for 5-year CSS in the development (0.621 vs. 0.609, p < 0.001) and internal validation cohorts (0.618 vs. 0.612, p = 0.016) and the npN was also better than the pN stage for 5-year CSS in the development (0.623 vs. 0.609, p < 0.001) and internal validation cohorts (0.620 vs. 0.612, p = 0.001). However, there was no significant difference between the AUCs of mpN and npN. Moreover, the pN stage for 5-year CSS in the external validation cohort is 0.606 vs. 0.563, p = 0.045.

**Conclusions:**

In stage III CC, mpN and npN may be superior to the pN stage in assessing prognosis, suggesting that the TD information should be included in the pN stage.

## Introduction

Globally, colon cancer (CC) is one of the most common cancers worldwide and the major causes of cancer-related mortality, forming a huge burden on both family and society (1, 2).The American Joint Commission on Cancer (AJCC) tumor-node-metastasis (TNM) classification is the most important factor in determining prognosis, which helps physicians make clinical decisions. Therefore, the TNM classification has been constantly modified and improved to enhance its ability to guide treatment and predict prognosis in recent years, particularly in terms of pathological N (pN) stage (3–5). Most notably, tumor deposits (TD) have always been a hot topic for medical experts and scholars.

TD are discrete foci of tumor in fat in the central (or perirectal) lymphatic drainage cavity of the primary tumor, with no histological evidence of vascular structure or residual lymph node tissue in the nodules (4, 6). TD are observed in ~20% of CC. A large number of studies have shown that TD is an independent prognostic factor for survival in CC patients. CC patients with TD+ had significantly shorter survival than those with TD- (7, 8). TNM classification clearly indicated that CC is classified as pN1c stage when regional lymph nodes are negative and TD are positive. However, when PLN were present, neither the status nor the number of TD was included in TNM classification. Hence, when regional lymph nodes were positive, the effect of TD on TNM classification is unclear. The 8th TNM classification requires the recording of the status and number of TD, although how to use it has not been agreed, which may affect the further TNM classification of CC. This study therefore aimed to comprehensively evaluate the prognostic role of TD in stage III CC and provide reference advice for the improvement of TNM classification.

## Methods

### Patients

This study included CC patients who were collected from the Surveillance, Epidemiology, and End Results (SEER) program and the Second Affiliated Hospital of Harbin Medical University between January 2010 and December 2015. Informed consent was not required because the SEER database is publicly available. Inclusion criteria included the following: 1) the pathological diagnosis was CC in stage III and underwent surgical treatment; 2) aged ≥ 18 years old; 3) patients with complete records of cancer-specific survival months and vital status; 4) CC was the only primary malignancy. Exclusion criteria included the following: 1) patient received neoadjuvant therapy; 2) patients without complete follow-up data; 3) the basic information of the patient is incomplete.

### Variables

According to our study, age was regrouped into ≤60 and >60 years old; sex was classified as male and female; and race was classified as white, black, and other. The tumor site was grouped into right colon and left colon. The histology variable was classified as “adenocarcinoma”, “mucinous”, or “others”; the grade variable was classified as “Well differentiated”, “Moderately differentiated”, “Poorly differentiated”, “Undifferentiated”, and unknown; and TD status was classified as “positive” and “negative”. Similarly, pT stage, pN stage, and chemotherapy are grouped. The carcinoembryonic antigen (CEA) level variable was classified as “positive” (≥5 ng/ml) and “negative” (<5 ng/ml).

### Statistical Analysis

All the statistical analyses were calculated in statistical software package SPSS 22.0 (IBM Corp, Armonk, NY, USA) and R software (version 3.6.1). Continuous variables were presented as mean ± standard deviation (SD). In order to increase the reliability of this article, patients were randomly (1:1 ratio) divided into development and validation cohorts. The clinical characteristics of patients were summarized by number and percentage. Kaplan–Meier analysis with log-rank tests was performed. The Cox proportional hazard regression model was used to identify independent prognostic factors. The area under the curve (AUC) was used to compare the discriminant ability of pN, mpN, and npN stages, with higher AUC demonstrating superior discrimination. All tests were two-sided, and a p value < 0.05 was considered statistically significant.

## Result

### Patient Characteristics

**Figure 1:**
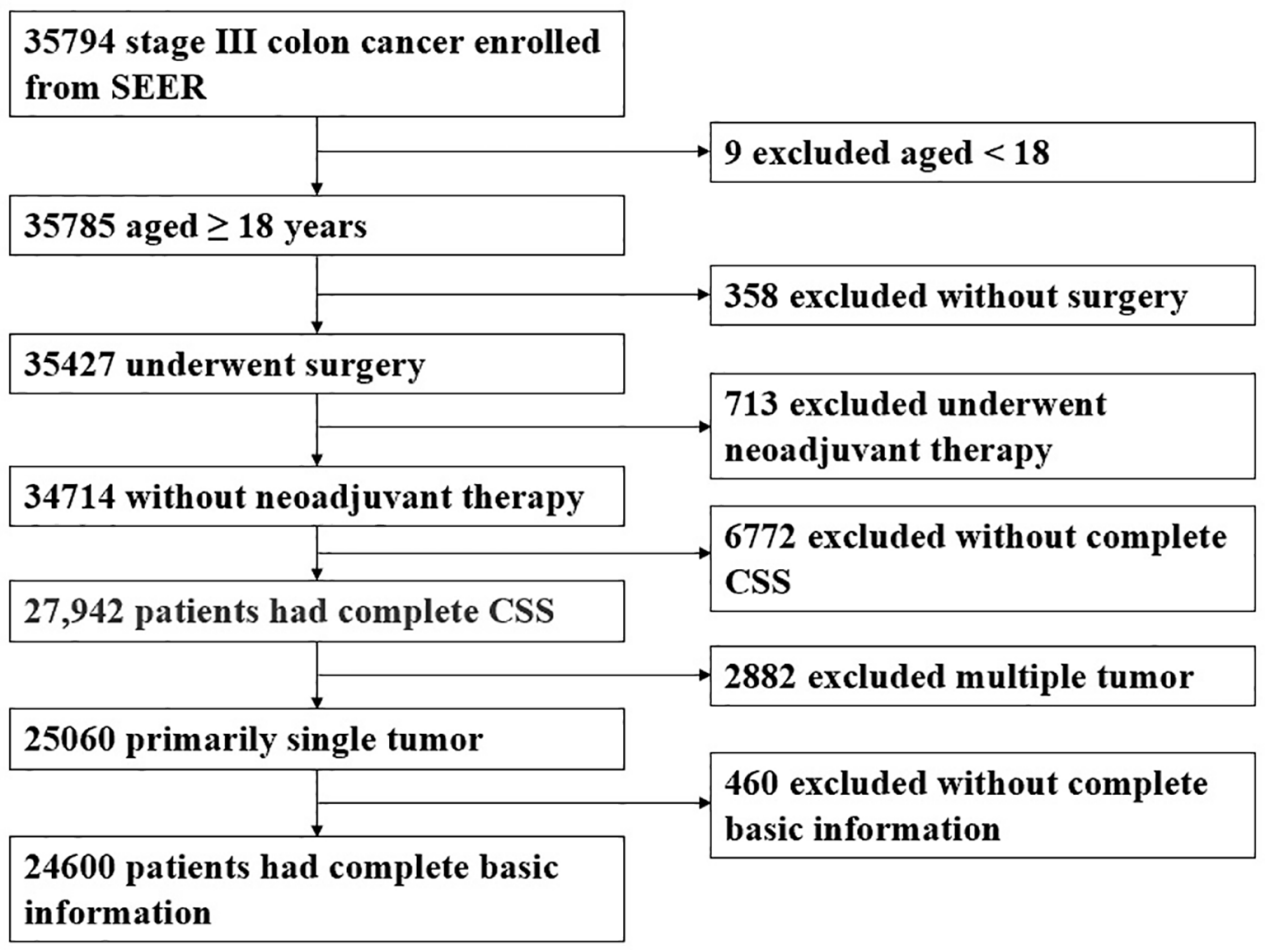
The patient inclusion process.

The patient inclusion process is shown in **Figure 1**. A total of 24,600 patients were evenly divided into the development and validation cohort in a 1:1 ratio. In the development cohort, females (51.2%), older than 60 years (64.8%) accounted for a higher proportion of patients. In 55.6% of colon cancer patients, the primary tumor site was in the right colon. The number of lymph nodes examined (LNE) was 20.6 ± 10.3, and the mean number of PLN was 3.4 ± 3.7. TD+ were observed in 1,864 cases (15.2%), and the mean number of TD was 0.4 ± 1.7. The clinicopathological characteristics of the validation cohorts were similar to the development cohort (**Table 1**).

**Table 1:** Characteristics of patients in the development and validation cohorts.

Characteristics	Development cohort (n = 12,300)	Internal validation cohort (n = 12,300)	p value	External validation cohort (n = 618)
**Age (years), n (%)**			0.670	
≤60	4,332 (35.2)	4,363 (35.5)		225 (36.4)
>60	7,968 (64.8)	7,936 (64.5)		393 (63.6)
**Race, n (%)**			0.738	
White	9,329 (75.8)	9,371 (76.2)		–
Black	1,601 (13.0)	1,596 (13.0)		–
Other	1,370 (11.1)	1,333 (10.8)		618 (100.0)
**Sex, n (%)**			0.320	
Male	6,003 (48.8)	6,081 (49.4)		309 (50.0)
Female	6,297 (51.2)	6,219 (50.6)		309 (50.0)
**Tumor site, n (%)**			0.158	
Right colon	6,839 (55.6)	6,949 (56.5)		333 (53.8)
Left colon	5,461 (44.4)	5,351 (43.5)		285 (46.2)
**Grade, n (%)**			0.091	
Well differentiated	610 (5.0)	590 (4.8)		36 (5.8)
Moderately differentiated	8,467 (68.8)	8,365 (68.0)		439 (71.1)
Poorly differentiated	2,563 (20.8)	2,624 (21.3)		114 (18.5)
Undifferentiated	503 (4.1)	580 (4.7)		24 (3.8)
Unknown	157 (1.3)	141 (1.1)		5 (0.8)
**Histology, n (%)**			0.616	
Adenocarcinoma	10,890 (88.5)	10,909 (88.7)		543 (87.8)
Mucinous	1,299 (10.6)	1,294 (10.5)		73 (11.8)
Other	111 (0.9)	97 (0.8)		2 (0.4)
**Chemotherapy**			0.519	
No	4,626 (37.6)	4,580 (37.2)		199 (32.2)
Yes	7,671 (62.4)	7,720 (62.8)		419 (67.8)
**CEA level, n (%)**			0.243	
Positive	3,168 (25.8)	3,208 (26.1)		161 (26.1)
Negative	4,414 (35.9)	4,501 (36.6)		278 (45.0)
Unknown	4,718 (38.4)	4,591 (37.3)		179 (28.9)
**pT, n (%)**			0.072	
T1	503 (4.1)	429 (3.5)		24 (3.9)
T2	1,180 (9.6)	1,128 (9.2)		67 (10.9)
T3	8,092 (65.8)	8,103 (65.9)		416 (67.4)
T4a	1,744 (14.2)	1,863 (15.1)		79 (12.7)
T4b	781 (6.3)	777 (6.3)		32 (5.1)
**pN, n (%)**			0.266	
N1a	4,020 (32.7)	4,046 (32.9)		216 (34.9)
N1b	3,973 (32.3)	3,829 (31.1)		179 (28.9)
N1c	448 (3.6)	455 (3.7)		22 (3.6)
N2a	2,252 (18.3)	2,276 (18.5)		113 (18.3)
N2b	1,607 (13.1)	1694 (13.8)		88 (14.3)
LNE^*^	20.6 ± 10.3	20.5 ± 10.0	0.485	20.8 ± 10.3
PLN*	3.4 ± 3.7	3.4 ± 3.8	0.494	3.5 ± 3.9
NTD*	0.4 ± 1.7	0.4 ± 1.9	0.764	0.4 ± 1.3
**TD status**			0.416	
Positive	1,864 (15.2)	1,910 (15.5)		508 (82.2)
Negative	10,436 (84.8)	10,390 (84.5)		110 (17.7)

LNE^*^, lymph nodes examined; PLN*, positive lymph nodes; NTD*, the number of the tumor deposits.

### Prognosis of TD

**Figure 2:**
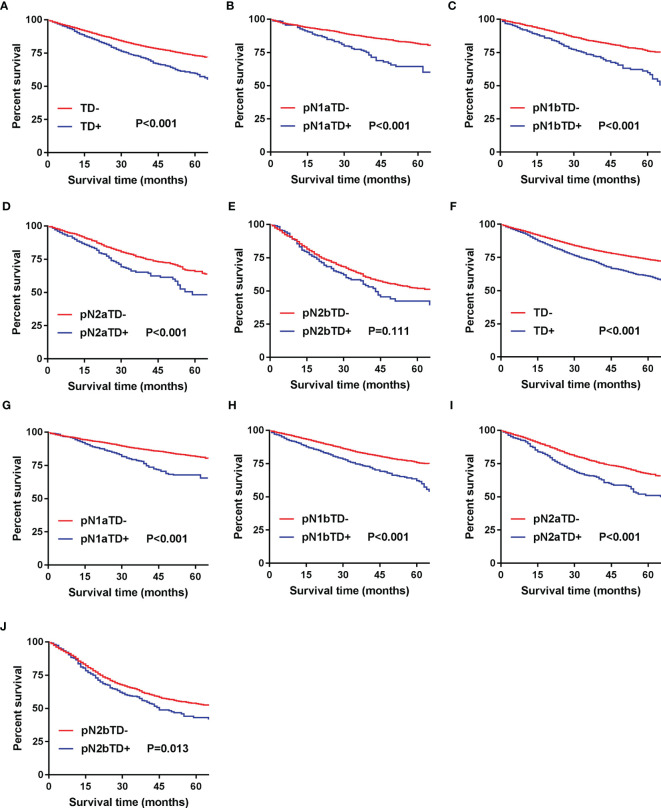
Kaplan–Meier survival curves for patients with and without TD among all patients and pN patients. **(A)** All patients in the development cohort; **(B)** pN1a substage in the development cohort; **(C)** pN1b substage in the development cohort; **(D)** pN2a substage in the development cohort; **(E)** pN2b substage in the development cohort; **(F)** all patients in the validation cohort; **(G)** pN1a substage in the validation cohort; **(H)** pN1b substage in the validation cohort; **(I)** pN2a substage in the validation cohort; **(J)** pN2b substage in the validation cohort.

In the development cohort, we found that 5-year CSS was significantly poorer in TD+ patients than in TD- patients (p < 0.001) (**Figure 2A**). The same analysis was done in stages pN1a, pN1b, pN2a, and pN2b, and similar results were found (**Figures 2B–E**). These results were confirmed in the validation cohort (**Figures 2F–J**).

### Construction of the Modified Stage (mpN) and Novel Stage (npN)

**Figure 3:**
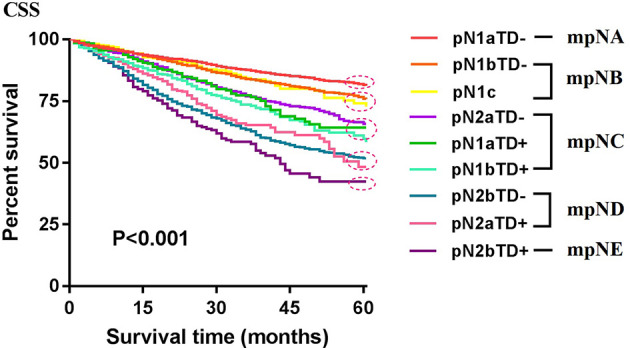
Kaplan–Meier survival analysis results showed the 5-year CSS trend among the 9 subgroups.

**Figure 4:**
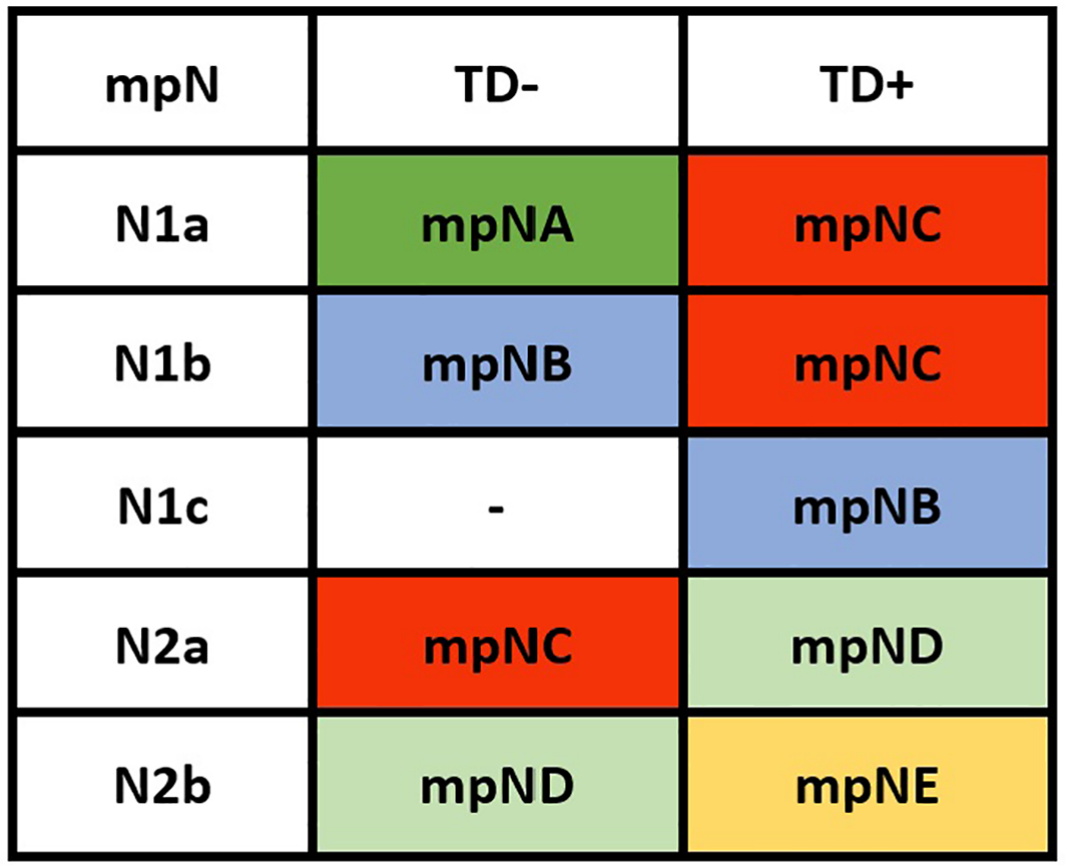
Modified pN stage (mpN).

Patients were redivided into 9 subgroups by combining the pN (pN1a, pN1b, pN1c, pN2a, and pN2b) with TD status (TD- and TD+): pN1aTD-, pN1aTD+, pN1bTD-, pN1bTD+, pN1c, pN2aTD-, pN2aTD+, pN2bTD-, and pN2bTD+. Kaplan–Meier survival analysis results showed the 5-year CSS trend among the 9 subgroups (**Figure 3**). Using pN1aTD- as a reference, all subgroups were redivided into five modified pN (mpN) based on the 5-year CSS rates and HRs (**Table 2**). The mpN include mpNA (pN1aTD-), mpNB (pN1bTD- and pN1c), mpNC (pN2aTD-, pN1aTD+, and pN1bTD+), mpND (pN2bTD- and pN2aTD+), and mpNE (pN2bTD+) (**Figure 4**). The 5-year CSS rates for mpNA, B, C, D, and E were 80.4%, 74.6%, 63.4%, 50.6%, and 40.8% in the development cohort (p < 0.001) and 80.6%, 74.2%, 67.3%, 53.3%, and 41.4% in the validation cohort, respectively (p < 0.001).

**Table 2:** Survival analysis among different subgroups in the development and validation cohorts.

Group	5-year CSS rate (%)	HR	95% CI	p
**Development cohort**				
pN1aTD-	80.4	1		
pN1aTD+	57.4	2.189	1.740-2.755	0.000
pN1bTD-	74.7	1.271	1.134-1.425	0.000
pN1bTD+	58.9	2.284	1.892-2.757	0.000
pN1c	73.1	1.287	1.011-1.639	0.041
pN2aTD-	64.7	1.827	1.616-2.064	0.000
pN2aTD+	47.9	2.719	2.192-3.373	0.000
pN2bTD-	50.6	3.079	2.728-3.476	0.000
pN2bTD+	37.7	4.004	3.292-4.868	0.000
**Validation cohort**				
pN1aTD-	80.8	1		
pN1aTD+	66.1	1.663	1.292-2.141	0.000
pN1bTD-	74.3	1.339	1.194-1.501	0.000
pN1bTD+	64.1	2.121	1.717-2.619	0.000
pN1c	73.6	1.366	1.076-1.735	0.010
pN2aTD-	67.6	1.761	1.555-1.994	0.000
pN2aTD+	51.8	3.062	2.519-3.721	0.000
pN2bTD-	53.6	3.105	2.748-3.508	0.000
pN2bTD+	41.4	4.000	3.321-4.818	0.000

Similarly, with TD considered as PLN, the number of PLN combined with the number of TD (PTCD) was used to construct the npN. In PLN cases, the conventional pN stage was divided mainly according to the number of PLN: pN1 (pN1a: 1 PLN; pN1b: 2-3 PLN; pN1c: TD formation) and pN2 (pN2a: 4–6 PLN; pN2a ≥ 7 PLN). In this study, we further modified this stage and construct the npN: npN1a (1 PCTD), npN1b: (2–3 PCTD), npN2a (4–6 PCTD), npN2b (≥7 PCTD).

### Univariate and Multivariate Survival Analyses

The univariate Cox proportional hazard regression model is shown in **Table 3**. In univariate analysis, the candidate predictors were age, race, tumor site, grade, histology, CEA level, chemotherapy, pT, pN, mpN, and npN. All the predictors except for sex were of statistical significance in the development cohort, which were then further analyzed by the multivariate Cox proportional hazard regression model (**Table 4**). Results showed that mpN and npN were independent prognostic factors for CSS in their respective study cohorts according to multivariate analysis.

**Table 3:** Univariate Cox regression analyses of factors related to CSS in the development cohort.

Characteristics	Univariate analysis	
	HR [95% CI]	p-value
**Age (years), n (%)**		
≤60	1	
>60	2.026 [1.845–2.214]	0.000
**Race, n (%)**		
White	1	
Black	1.049 [0.939–1.172]	0.398
Other	0.821 [0.721–0.936]	0.003
**Sex, n (%)**		
Male	1	
Female	1.065 [0.988–1149]	0.101
**Tumor site, n (%)**		
Right colon	1	
Left colon	0.645 [0.596–0.698]	0.000
**Grade, n (%)**		
Well differentiated	1	
Moderately differentiated	0.976 [0.808–1.178]	0.800
Poorly differentiated	1.729 [1.422–2.102]	0.000
Undifferentiated	2.354 [1.867–2.967]	0.000
Unknown	1.461 [1.036–2.036]	0.031
**Histology, n (%)**		
Adenocarcinoma	1	
Mucinous	1.388 [1.241–1.552]	0.000
Other	2.008 [1.457–2.766]	0.000
**CEA level, n (%)**		
Positive	1	
Negative	1.877 [1.701–2.071]	0.000
Unknown	1.525 [1.389–1.675]	0.000
**Chemotherapy**		
No	1	
Yes	0.376 [0.357–0.397]	0.000
**pT, n (%)**		
T1	1	
T2	1.440 [0.963–2.155]	0.076
T3	3.673 [2.574–5.240]	0.000
T4a	7.551 [5.264–10.833]	0.000
T4b	9.331 [6.455–13.487]	0.000
**mpN, n (%)**		
A	1	
B	1.273 [1.139–1.423]	0.000
C	1.939 [1.734–2.169]	0.000
D	3.014 [2.684–3.385]	0.000
E	4.002 [3.292–4.867]	0.000
**npN, n (%)**		
1a	1	
1b	1.329 [1.194–1.481]	0.000
2a	1.884 [1.682–2.111]	0.000
2b	3.269 [2.932–3.645]	0.000

**Table 4:** Multivariate Cox regression analyses of factors related to CSS in the development cohort.

Characteristics	Multivariate analysis (mpN)		Multivariate analysis (npN)	
	HR [95% CI]	p-value	HR [95% CI]	p-value
**Age (years), n (%)**				
≤60	1		1	
>60	1.604 [1.461–1.761]	0.000	1.617 [1.472–1.775]	0.000
**Race, n (%)**				
White	1		1	
Black	1.189 [1.062–1.330]	0.003	1.204 [1.076–1.347]	0.001
Other	0.837 [0.735–0.955]	0.008	0.840 [0.737–0.958]	0.009
**Tumor site, n (%)**				
Right colon	1		1	
Left colon	0.827 [0.762–0.897]	0.000	0.830 [0.765–0.901]	0.000
**Grade, n (%)**				
Well differentiated	1		1	
Moderately differentiated	0.949 [0.785–1.146]	0.584	0.950 [0.786–1.147]	0.594
Poorly differentiated	1.260 [1.035–1.534]	0.021	1.257 [1.033–1.531]	0.022
Undifferentiated	1.614 [1.276–2.042]	0.000	1.603 [1.267–2.208]	0.001
Unknown	1.159 [0.820–1.637]	0.460	1.139 [0.806–1.609]	0.460
**Histology, n (%)**				
Adenocarcinoma	1		1	
Mucinous	1.074 [0.959–1.204]	0.216	1.058 [0.944–1.186]	0.331
Other	1.192 [0.859–1.654]	0.294	1.188 [0.856–1.649]	0.302
**CEA level, n (%)**				
Positive	1			
Negative	1.462 [1.323–1.615]	0.000	1.456 [1.318–1.608]	0.000
Unknown	1.255 [1.142–1.380]	0.000	1.253 [1.140–1.377]	0.000
**Chemotherapy**				
No	1		1	
Yes	0.400 [0.369–0.432]	0.000	0.401 [0.370–0.434]	0.000
**pT, n (%)**				
T1	1		1	
T2	1.253 [0.837–1.875]	0.274	1.265 [0.845–1.893]	0.254
T3	2.596 [1.816–3.710]	0.000	2.630 [1.841–3.758]	0.000
T4a	4.819 [3.350–6.931]	0.000	4.873 [3.388–7.008]	0.000
T4b	5.624 [3.879–8.154]	0.000	5.670 [3.911–8.220]	0.000
**mpN, n (%)**				
A	1		–	–
B	1.228 [1.098–1.373]	0.000	–	–
C	1.856 [1.657–2.077]	0.000	–	–
D	2.620 [2.326–2.952]	0.000	–	–
E	3.484 [2.857–4.249]	0.000	–	–
**npN, n (%)**				
1a	–	–	1	
1b	–	–	1.310 [1.176–1.459]	0.000
2a	–	–	1.841 [1.642–2.065]	0.000
2b	–	–	2.867 [2.563–3.208]	0.000

### Five-Year CSS in Relation to pN, mpN, and npN

**Figure 5:**
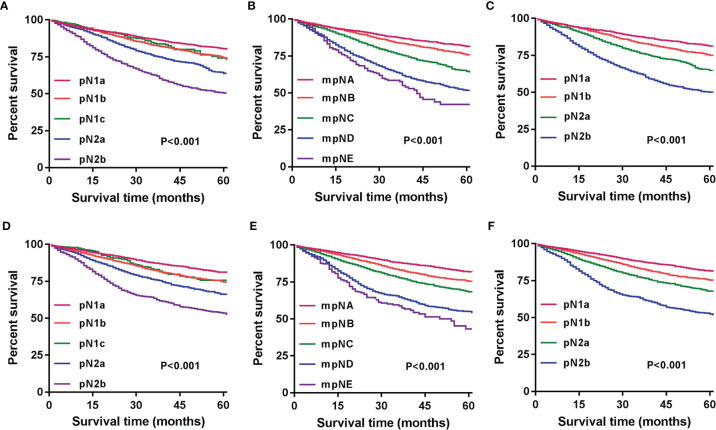
Kaplan–Meier survival curves for 5-year CSS based on the pN, mpN, and npN stages. Kaplan–Meier survival curves based on **(A)** pN stage in the development cohort; **(B)** mpN stage in the development cohort; **(C)** npN stage in the development cohort; **(D)** pN stage in the internal validation cohort; **(E)** mpN stage in the internal validation cohort; and **(F)** npN stage in the internal validation cohort.

The 5-year CSS of patients with stages pN1a, pN1b, pN1c, pN2a, and pN2b were 79.1%, 73.1%, 73.1%, 62.9%, and 49.1% in the development cohort (p < 0.001) and 79.9%, 73.4%, 73.6%, 65.3%, and 51.6% in the validation cohort, respectively (p < 0.001) (**Figures 5A, D**). We could find that the 5-year CSS of pN1b and pN1c stages are very similar, and there was no significant difference between the two nodal stages (development cohort: p = 0.459; validation cohort: p = 0.284). The mpN showed enhanced stratification to differentiate between all substages (p < 0.001; **Figure 5B**). Similar results were found in the validation cohort (**Figure 5E**).

**Figure 6:**
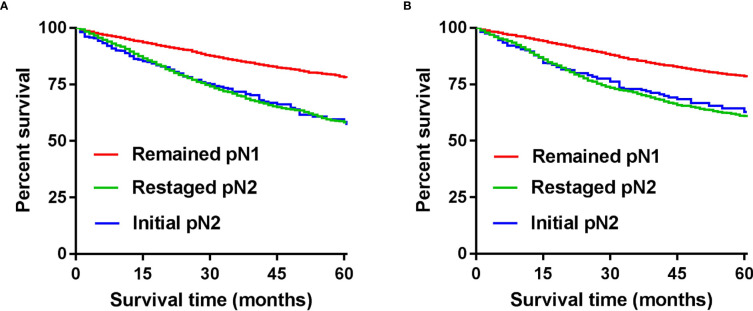
CSS according to the number of TD and by npN stage after adding TD to the number of PLN in the development **(A)** and internal validation cohort **(B)**.

We combined the number of PLN with the number of TD to form the npN, which treated the TD as PLN, and the KM curves of 5-year CSS show good differentiation than the pN stage in the development (**Figure 5C**) and validation cohorts (**Figure 5F**). It is noteworthy that part of the pN1 stage is bound to be restaged as the pN2 stage with the application of the npN. Survival analysis showed that restaged pN2 has a significant difference with remained pN1, but there was no statistical difference with initial pN2 (**Figure 6A**). Similar results were found in the validation cohort (**Figure 6B**).

### Superiority of the mpN and npN

**Figure 7:**
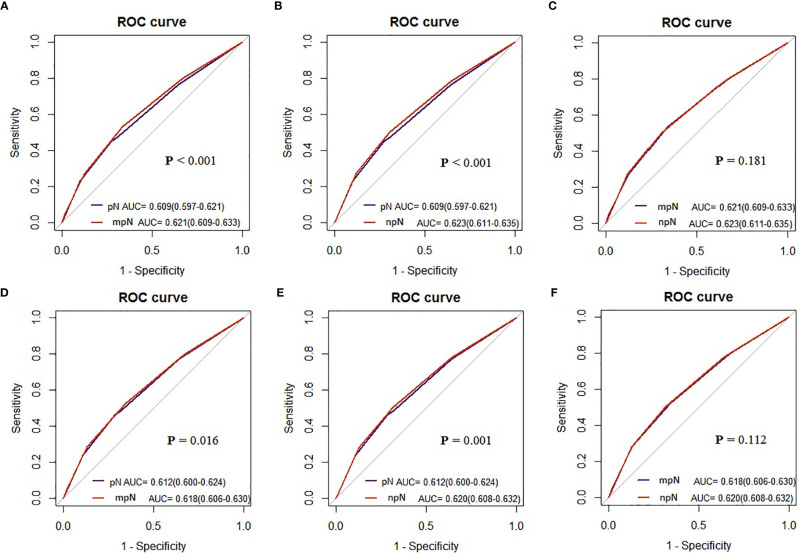
The AUCs of the pN, mpN, and npN stage in the development **(A–C)** and internal validation cohorts **(D–F)**.

**Figure 8:**
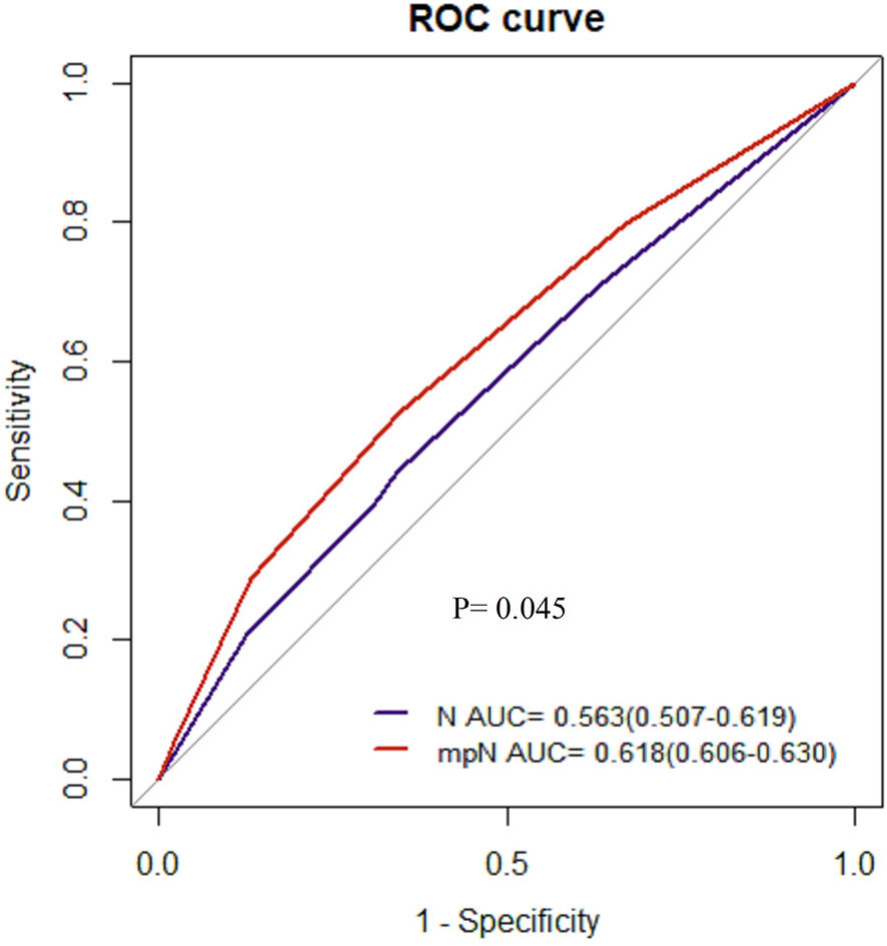
The AUCs of the pN and mpN stage in the external validation cohort.

AUCs of the mpN and npN at 5-year CSS indicated the better discrimination ability compared to the pN stage. The AUCs of the mpN and pN stages at 5-year CSS were 0.621 (95% CI = 0.609–0.633) and 0.609 (95% CI = 0.597–0.621) in the development cohort (p < 0.001) (**Figure 7A**) and 0.618 (95% CI = 0.606–0.630) and 0.612 (95% CI = 0.600–0.624) in the validation cohort (p = 0.016) (**Figure 7D**). Moreover, the AUCs of the npN and pN stages at 5 years were 0.623 (95% CI = 0.611–0.635) and 0.609 (95% CI = 0.597–0.621) in the development cohort (p < 0.001) (**Figure 7B**) and 0.620 (95% CI = 0.608–0.632) and 0.612 (95% CI = 0.600–0.624) in the validation cohort (p = 0.001) (**Figure 7E**). However, there was no significant difference in AUC between mpN and npN in the development and validation cohorts (**Figures 7C, F**). Moreover, the predictive accuracy of mpN was better than that of the pN stage for 5-year CSS in the external validation cohort (0.618 vs. 0.563, p = 0.045) (**Figure 8**).

## Discussion

The AJCC TNM classification of CC is the most important factor in managing patients and determining prognosis. TD refers to a discrete nodule of cancer in pericolic/perirectal fat or adjacent mesentery without identifiable lymph node tissue or vascular structure which are observed in ~20% of CC. The relationship between TD and PLN has long been a topic of debate among experts and scholars, resulting in three revisions to the guidelines for the diagnosis and treatment of CC. The AJCC 5th TNM classification defined TD based on the maximum diameter: nodules < 3 mm were classified as TD and nodules ≥ 3 mm as PLN (3). The AJCC 6th TNM classification defined TD based on their contours: irregularly contoured nodules were regarded as TD, while regular smooth nodules were regarded as positive PLN. The AJCC 7th TNM classification incorporated TD into the TNM staging and defined any pT TD+ and regional lymph nodes are negative as pN1c. The AJCC 8th TNM classification remains unchanged (6).

There is no denying that TD+ is strongly associated with poor prognosis in CC patients (9–13). Some studies have shown that TD+ is also significantly correlated with local recurrence and distant metastasis (8, 12), and the higher the number of TD, the worse the prognosis of CC patients. In conclusion, the status and number of TD are closely related to the prognosis of CC patients. However, The AJCC 8th TNM classification clearly states that TD should only be included in staging if regional lymph nodes are negative. Obviously, it is not reasonable to ignore TD when regional lymph nodes are positive. Therefore, the main purpose of this study is to explore what role TD should play in TNM classification in the presence of PLN.

Iris D. Nagtegaal et al. (14) indicated that TD’s origins are varied; almost 40% showed a combined perineural, perivascular, and intravascular origin. A perineural origin was present in 77% of cases and an intravascular origin in 83% of cases. Basnet et al. (15) have reached similar conclusions without providing detailed information on the exact role of TD. The presence or absence of TD in TNM staging was considered as a priority (16). In this study, we classified stage III CC according to TD status, and the results showed that among all pN stages, the 5-year CSS of patients with TD+ was significantly lower than that of patients with TD-. These results suggest that TD status should be considered as a potential poor prognostic factor in CC patients.

Moreover, some studies have suggested that TD can be considered as PLN or metastatic disease, even though TD are not PLN per se (17, 18). Jin et al. (11) and Lin et al. (19) reported that the increased number of TD was associated with poor prognosis. They concluded that the number of TD also affects prognosis and that further risk classification based on the number of TD is feasible. In the npN of this study, TD was considered to be PLN and the number of regional lymph node metastases was considered to be the total number of PLN and TD. It is noteworthy that the KM curve showed that restaged pN2 has a significant difference with remained pN1, but there was no statistical difference with initial pN2 with the application of the npN. Peilin Zhang et al. (20) reached a view consistent with this study after undergoing PSM of patients. Accordingly, the number of PLN combined with the number of TD is a feasible strategy.

Finally, we included the status and number of TD in the mpN and npN. A good classification system should show prognostic discrimination where the survival analysis of each group should be significantly different. Survival analysis showed that the mpN and npN had better discrimination ability compared with the pN stage. Moreover, AUC analysis also showed that mpN and npN had higher discriminating and model-fitting abilities. Although there was no significant difference in AUC between mpN and npN, npN has a higher AUC than mpN.

There are several innovations in our research. First of all, this study establishes mpN and npN respectively with the same population and obtains convincing results. Then, we compared mpN with npN, and npN has a higher AUC. Finally, we did a validation cohort to make our results more convincing.

This study has several limitations. Firstly, this was a large-scale retrospective study, and the lack of rigorous experimental design may have led to selection bias. However, the sample size is large, which may reduce this risk. Secondly, although this study has validation based on the Chinese population, it may cause errors due to insufficient data from external validation. The external validation cohort lacks the database of the number of TD to further validate. Thirdly, only cancer-specific survival was discussed in this study, and overall survival was not discussed because the authors believed that overall survival was more confounding. Fourth, although our study confirmed the effect of TD on the survival of CC patients and clarified how to use it to judge the prognosis, the mechanism of the effect is still unclear.

In conclusion, among patients with CC and LN metastases, mpN or npN may be superior to the conventional pN stage in assessing prognosis, suggesting that the status or number of TD should be included in the pN stage.

## Data Availability Statement

The original contributions presented in the study are included in the article/supplementary material. Further inquiries can be directed to the corresponding author.

## Ethics Statement

This study received ethical approval from the Second Affiliated Hospital of Harbin Medical University. The study used de-identified data and adhered to the World Medical Association’s Declaration of Helsinki for Ethical Human Research. SEER is a publicly available database with anonymized data; no ethical review was required.

## Author Contributions

All authors made a significant contribution to the work reported, whether that is in the conception, study design, execution, acquisition of data, analysis and interpretation, or in all these areas; took part in drafting, revising, or critically reviewing the article; gave final approval of the version to be published; have agreed on the journal to which the article has been submitted; and agree to be accountable for all aspects of the work.

## Conflict of Interest

The authors declare that the research was conducted in the absence of any commercial or financial relationships that could be construed as a potential conflict of interest.

## Publisher’s Note

All claims expressed in this article are solely those of the authors and do not necessarily represent those of their affiliated organizations, or those of the publisher, the editors and the reviewers. Any product that may be evaluated in this article, or claim that may be made by its manufacturer, is not guaranteed or endorsed by the publisher.
